# Effect of Polyhydroxybutyrate (PHB) storage on l-arginine production in recombinant *Corynebacterium crenatum* using coenzyme regulation

**DOI:** 10.1186/s12934-016-0414-x

**Published:** 2016-01-19

**Authors:** Meijuan Xu, Jingru Qin, Zhiming Rao, Hengyi You, Xian Zhang, Taowei Yang, Xiaoyuan Wang, Zhenghong Xu

**Affiliations:** The Key Laboratory of Industrial Biotechnology of Ministry of Education, School of Biotechnology, Jiangnan University, Wuxi, 214122 Jiangsu People’s Republic of China; Laboratory of Pharmaceutical Engineering, School of Medicine and Pharmaceutics, Jiangnan University, Wuxi, 214122 Jiangsu People’s Republic of China

**Keywords:** l-Arginine, Poly-β-hydroxybutyrate (PHB), NAD kinase, *Corynebacterium crenatum* SYPA 5

## Abstract

**Background:**

*Corynebacterium crenatum* SYPA 5 is the industrial strain for l-arginine production. Poly-β-hydroxybutyrate (PHB) is a kind of biopolymer stored as bacterial reserve materials for carbon and energy. The introduction of the PHB synthesis pathway into several strains can regulate the global metabolic pathway. In addition, both the pathways of PHB and l-arginine biosynthesis in the cells are NADPH-dependent. NAD kinase could upregulate the NADPH concentration in the bacteria. Thus, it is interesting to investigate how both PHB and NAD kinase affect the l-arginine biosynthesis in *C. crenatum* SYPA 5.

**Results:**

*C. crenatum* P1 containing PHB synthesis pathway was constructed and cultivated in batch fermentation for 96 h. The enzyme activities of the key enzymes were enhanced comparing to the control strain *C. crenatum* SYPA 5. More PHB was found in *C. crenatum* P1, up to 12.7 % of the dry cell weight. Higher growth level and enhanced glucose consumptions were also observed in *C. crenatum* P1. With respect to the yield of l-arginine, it was 38.54 ± 0.81 g/L, increasing by 20.6 %, comparing to the control under the influence of PHB accumulation. For more NADPH supply, *C. crenatum* P2 was constructed with overexpression of NAD kinase based on *C. crenatum* P1. The NADPH concentration was increased in *C. crenatum* P2 comparing to the control. PHB content reached 15.7 % and 41.11 ± 1.21 g/L l-arginine was obtained in *C. crenatum* P2, increased by 28.6 %. The transcription levels of key l-arginine synthesis genes, *arg*B*, arg*C*, arg*D and *arg*J in recombinant *C. crenatum* increased 1.9–3.0 times compared with the parent strain.

**Conclusions:**

Accumulation of PHB by introducing PHB synthesis pathway, together with up-regulation of coenzyme level by overexpressing NAD kinase, enables the recombinant *C. crenatum* to serve as high-efficiency cell factories in the long-time l-arginine fermentation. Furthermore, batch cultivation of the engineered *C. crenatum* revealed that it could accumulate both extracellular l-arginine and intracellular PHB simultaneously. All of these have a potential biotechnological application as a strategy for high-yield l-arginine.

**Electronic supplementary material:**

The online version of this article (doi:10.1186/s12934-016-0414-x) contains supplementary material, which is available to authorized users.

## Background

l-Arginine is a kind of semi-essential amino acid and plays a significant role in nitrogen metabolism and ammonia detoxification as an intermediate in the urea cycle in humans [1]. It is involved in numerous areas of application, such as food flavor, pharmacology and physiology [2, 3]. The biosynthesis of l-arginine in bacteria has become a focus of research interest for the past decades on metabolic regulation. Studies on the l-arginine production have been conducted using the mutants of *Corynebacterium*, *Bacillus* and *Serratia* since the 1960s [4–6]. Several elaborate strategies were designed for efficient production of L-arginine based on the pathways, regulation, and metabolic reaction of amino acids [7, 8]. *Corynebacterium crenatum* was successfully isolated from soil, and its mutated strain, *C. crenatum* SYPA 5, is an aerobic, gram-positive, non-sporulating and l-histidine auxotroph industrial bacterium [9, 10]. In our previous work, much work has been done to increase the production of l-arginine using *C. crenatum* SYPA 5 as the start strain by adjusting the transfer efficiency for l-arginine, increasing the dissolved oxygen in bacterial, modifying the key enzymes involved in the l-arginine synthesis pathway [5, 10–12].

Poly-β-hydroxybutyrate (PHB), stored as bacterial reserve materials for carbon and energy, is the most popular type of polyhydroxyalkanoate (PHA) that has been well studied in recent years [13]. It is an environmentally friendly biopolymer material due to its prominent properties, such as biodegradability and biocompatibility [14, 15]. PHB is intracellular while l-arginine is extracellular. It is possible to produce l-arginine and PHB simultaneously, which improves the resource utilization rate. In addition, PHB can provide the cells with carbon source, energy and reducing power, which influence intracellular metabolic flow, oxidation/reduction state and enhance stress resistance of the cells [16]. As reported, the production of several amino acids was enhanced resulting from the extra introduction of the PHB synthesis pathway. In some way, the accumulation of PHB in the cells could be seen as a strategy for amino acids and important metabolic compounds production [17–19]. More the effect of PHB accumulation in the bacterial on l-arginine yield has not been reported yet. During the PHB biosynthesis process, three key enzymes exist including PHB synthase (PhbC), β-ketothiolase (PhbA) and NADPH-dependent acetoacetyl-CoA reductase (PhbB). Among them, PhbB is quite special and plays a significant role in PHB synthesis due to its dependence upon NADPH [20]. A high level of NADPH and/or NADPH/NADP^+^ ratio has a critical effect on PHB synthesis [21].

During the l-arginine biosynthesis process, the cofactor, like the NADPH concentration, is known to have an important influence on the production by microorganisms because those key enzymes involved in l-arginine biosynthesis require NADPH, such as the NADPH-dependent glutamate dehydrogenase (GdhA) and ArgC [22]. However, high-yielded l-arginine by enhancing the coenzyme level in the cells has not drawn much attention.

The cofactor pairs NADPH/NADP^+^ is essential for all living organisms and plays its important role, mainly in its use as donor and/or acceptor of reducing equivalents in oxidation–reduction reactions in living cells [23]. Many industrially valuable compounds require NADPH for their synthesis and there have been a variety of methods designed to (re)generate this cofactor, like chemical, electrochemical, photochemical, or enzymatic reactions [24, 25]. NADPH can be generated by phosphorylating NAD through NAD kinase. NAD kinase catalyses NAD phosphorylation using ATP and/or inorganic polyphosphate [poly(P)] as phosphoryl donors in the presence of Mg^2+^ [26]. NAD kinase is ubiquitously distributed from bacteria to human cells and the gene encoding NAD kinase in *Escherichia coli*, *Saccharomyces cerevisiae*, and humans have been identified and well-studied [27–30]. Changing the cofactor level by overexpressing the NAD kinase has a positive effect on many industrially valuable compounds, like l-isoleucine in *C. glutamicum*, isobutanol and thymidine in *E. coli* [31–33]. As reported, *ppnK* was the only NAD kinase gene in *C. glutamicum* and PpnK is essential in the bacteria [34].

Both synthesis pathways of extracellular l-arginine and intracellular PHB are NADPH-dependent. They compete for NADPH, to some degree. Therefore, measures must be taken to improve the cofactor level. It is interesting to see the effect of NAD kinase overexpression on the PHB and l-arginine yield in *C. crenatum*, together with the effect of PHB on l-arginine yield. In this study, we were intended to construct a high-yield l-arginine strain by introducing the PHB synthesis pathway using *C. crenatum* SYPA 5 as the start strain. Meanwhile, the NAD kinase, PpnK, was overexpressed to balance the cofactor level. Furthermore, batch cultivation of the engineered *C. crenatum* revealed that it was able to accumulate both extracellular l-arginine and intracellular PHB simultaneously.

## Results and discussion

### Construction of *C. crenatum* P1 and *C. crenatum* P2

**Fig. 1:**
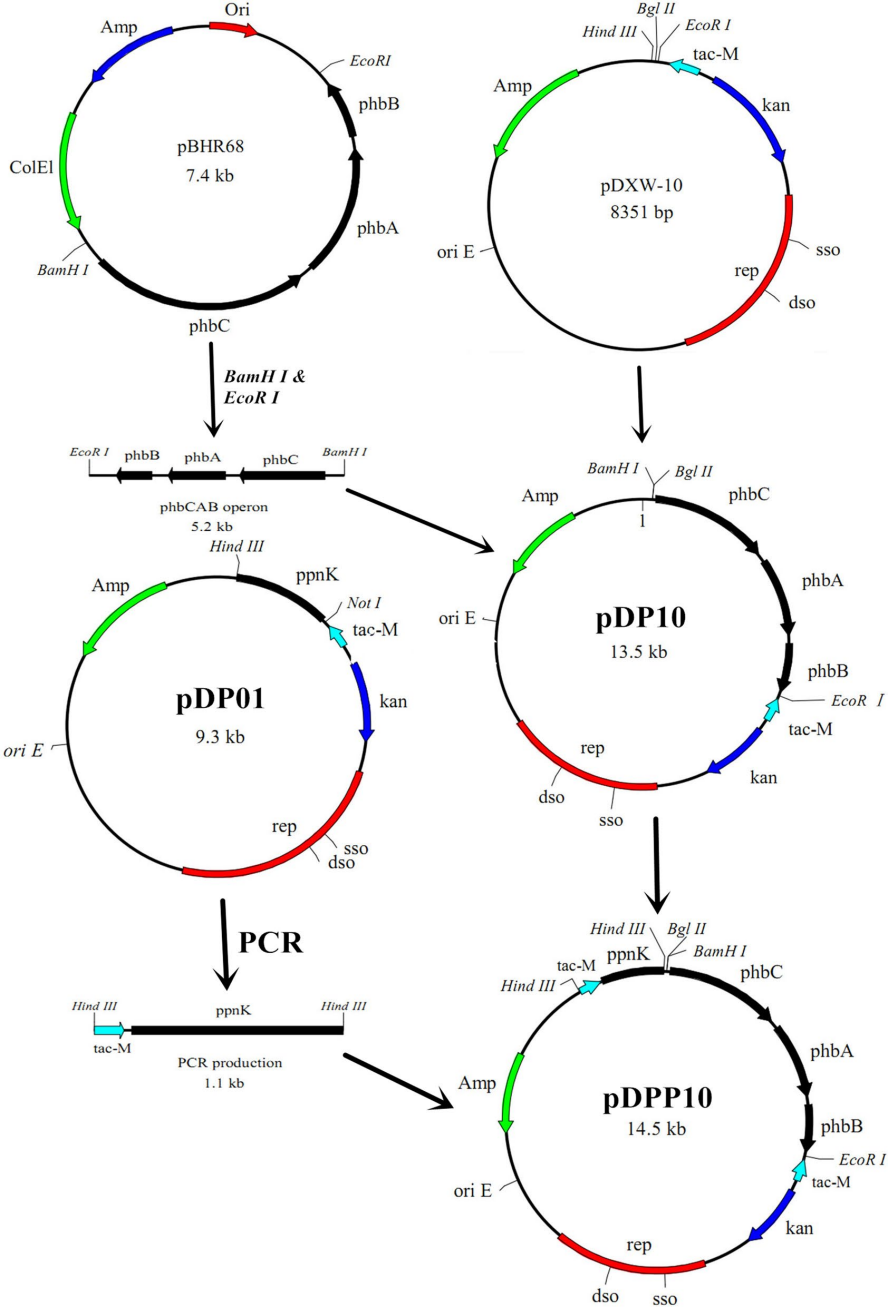
Construction of the plasmids in this study

**Fig. 2:**
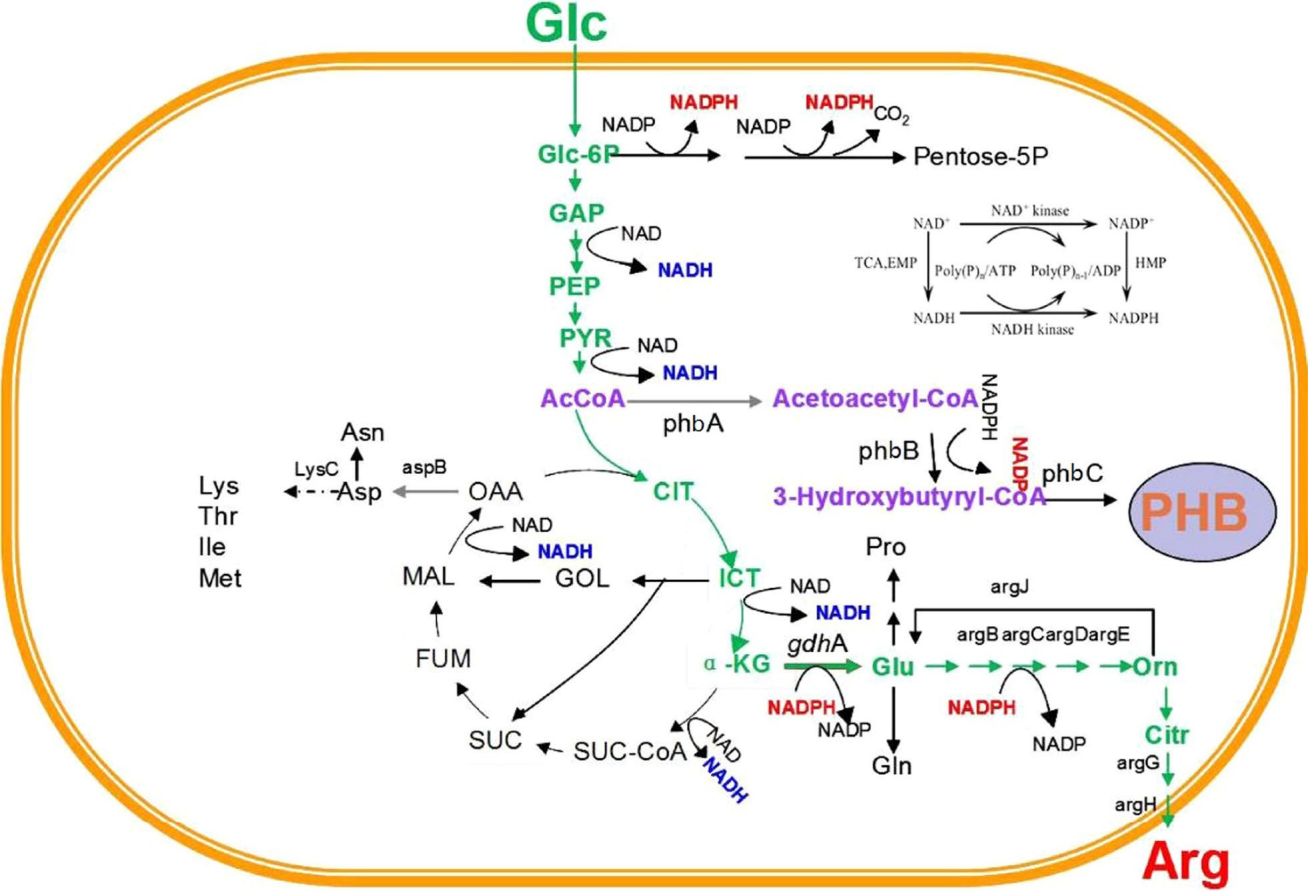
The metabolic pathways for the production of l-arginine and PHB in recombinant *C. crenatum*

PHB, the best-known polyhydroxyalkanoates (PHA), has been reported to influence intracellular metabolic flow, oxidation/reduction state [18] and enhance stress resistance of the host [16], as well. In this study, pDP10, containing the PHB synthesis genes, *phbCAB*, from *Ralstonia eutropha*, was introduced into *C. crenatum* SYPA 5 to generate *C. crenatum* P1 (Fig. 1). As depicted in Fig. 2, l-arginine metabolic pathway started from acetyl-CoA to α-ketoglutarate through TCA cycle, and then L-glutamate was formed with GdhA. Finally, l-arginine was produced by the catalysis of a series of enzymes encoded by the *arg*CJBDFRJH (*arg*C~H) cluster involved in l-arginine biosynthesis. On the other hand, PHB was formed from acetyl-CoA with the three key enzymes, PHB synthase (PhbC), β-ketothiolase (PhbA) and NADPH-dependent acetoacetyl-CoA reductase (PhbB).

Sources of NADPH in bacteria mainly contain the pentose phosphate pathway (PPP), isocitrate dehydrogenase in the tricarboxylic acid (TCA) cycle and the transhydrogenase system [23]. As illustrated in Fig. 2, NADPH was necessary in both the l-arginine and PHB metabolic pathways. PHB synthesis was a NADPH-dependent process for PhbB worked under the existence of NADPH and during the l-arginine metabolic pathways, GdhA and ArgC also required NADPH. In addition, in previous studies with arginine fermentation in *C. crenatum* SYPA 5, too much NADH can reduce the metabolic flow of the glycolytic pathway and increase the by-products (lactate and acetate) concentration [9]. As reported, in *C. glutamicum*, the NAD kinase (PpnK) regulated the cofactor level and could enhance the NADPH concentration [34]. Differential expression of *ppnK* has not been reported to date and *ppnK* might be essential and the only NAD kinase gene in *Corynebacterium sp.* [35]. Therefore, overexpressing homologous NAD kinase by cloning and amplifying the homologous *ppnK* gene in *C. crenatum* P1 deserved an attempt. We developed an approach to increase the NADPH availability in vivo through introducing NAD kinase, the key NADPH producing enzyme in *C. crenatum*, and were intended to see whether it had any effect on the l-arginine and PHB production or not. *C. crenatum* P2, containing the pDPP10, was created by introducing PpnK on the basis of *C. crenatum* P1.

### Enzyme activities assay of PhbC, PhbA, PhbB and PpnK

In this study, the *phbCAB* cluster of *Ralstonia eutropha* was introduced into *C. crenatum* SYPA 5. In order to verify the activity of the over-expressed PhbC, PhbA and PhbB from the *C. crenatum* PHB production strains, *C. crenatum* SYPA 5, *C. crenatum* P1 and *C. crenatum* P2 were cultivated in LBG medium (LB with 0.5 % glucose) for 24 h and then the crude enzyme activities of PhbC, PhbA and PhbB were detected (Table 1). The synthesis of bacterial PHB was dependent on the expression and activity of a key enzyme, PHB synthase (PhbC). Therefore, enhancing the activity of PhbC was a good way to increase PHB content [36]. To investigate the activity of PhbC, 3HB-CoA was used as the substrate, and the release of CoA during polymerization was measured to determine the total enzyme activity [37]. The total activity of PhbC was measured using the soluble fraction of the crude extract. The total synthase activity of cell extracts containing PhbC in recombinant *C. crenatum* was about 0.28 U/mg while it was quite low, 0.02 U/mg, in *C. crenatum* SYPA 5. As the first enzyme in the PHB synthesis pathway, the regulatory role of β-ketothiolase (PhbA) has been extensively discussed. PhbA from different strains differed with respect to the effect of concentrations of acetoacetyl-CoA or NADH and NADPH on the thiolysis reaction [38]. The enzyme activities of PhbA in *C. crenatum* P1 and *C. crenatum* P2 showed a significant increase, 30-fold, compared to the *C. crenatum* SYPA 5. The effect of about 11-fold enhancement in recombinant *C. crenatum* was found in the activities of NADPH-dependent acetoacetyl-CoA reductase (PhbB). PhbB was special among the three key PHB synthesis enzymes due to its coenzyme dependency upon NADPH. Therefore, the concentration of NADPH has quiet influence on the *phbB* expression. The high activity of PHB synthesis related enzymes in recombinant strains could be associated with their elevated level of genes expression through introducing the exogenous plasmid pDP10 or pDPP10.

**Table 1:** Assay of enzyme activities of crude PhbC, PhbA, PhbB and PpnK in *C. crenatum* SYPA 5, P1 (SYPA 5/phbCAB) and P2 (SYPA 5/phbCAB-ppnK)

Strains	Specific enzyme activities				
	PhbC (U/mg)	PhbA (U/mg)	PhbB (U/mg)	ATP-NAD kinase (U/g)	PolyP-NAD kinase (U/g)
*C. crenatum* SYPA 5	0.02 ± 0.00	0.02 ± 0.00	0.06 ± 0.01	0.63 ± 0.03	0.18 ± 0.01
*C. crenatum* P1	0.27 ± 0.01	0.58 ± 0.02	0.64 ± 0.02	0.67 ± 0.04	0.20 ± 0.01
*C. crenatum* P2	0.29 ± 0.01	0.61 ± 0.03	0.83 ± 0.03	84.35 ± 0.41	3.12 ± 0.02

Samples were taken at 24 h of the shake flask using LBG culture. ATP-NAD kinase contained ATP-NAD^+^ kinase and ATP-NADH kinase while PolyP-NAD kinase contained PolyP-NAD^+^ kinase and PolyP-NADH kinase. Each data represented the average value of three independent measurements

NAD kinase, PpnK, was the key enzyme for the biosynthesis of NADP^+^ and NADPH in *C. crenatum* and it was critical for the generation of NADPH [39]. It could be divided into two kinds according to the phosphoryl acceptor. The enzyme that phosphorylates only NAD^+^ to form NADP^+^ was termed NAD^+^ kinase (EC 2.7.1.23), and the enzyme that phosphorylates both NAD^+^ and NADH to form NADP^+^ and NADPH is NADH kinase (EC 2.7.1.86) [40, 41]. So far, the NAD kinases characterized either use ATP and PolyP as phosphoryl donors or were solely active with ATP [34] and PpnK here was the former kind. In order to investigate whether the *ppnK* gene expressed well in *C. crenatum* P2, the NAD kinase activity was determined and compared to the control *C. crenatum* SYPA 5. In the crude cell extract from the *ppnK*-expressing strain, the ATP-dependent and PolyP-dependent NAD kinase activity increased approximately 130-fold (84.35 ± 0.41 U/g) and 16-fold (3.12 ± 0.02 U/g), respectively, compared to the control *C. crenatum* SYPA 5 (0.63 ± 0.03 U/g) and (0.18 ± 0.01 U/g) (Data showed in Additional file 1). From the above observations, these differences in enzyme activities reflected the different expression levels of the PpnK proteins in these microorganisms, which might be due to the existence of the strong *tac*-M promoter [42].

### The effect of PHB accumulation on l-arginine operon transcription

**Fig. 3:**
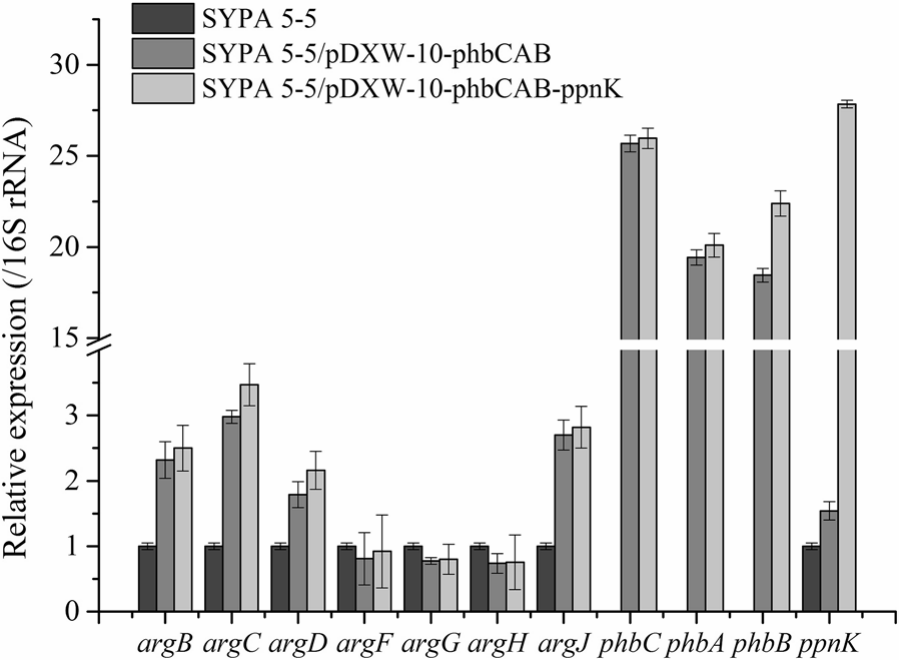
RT-qPCR analysis of key genes in the biosynthetic pathway of l-arginine and PHB. The mRNA expression level of the *arg*C~H, *phb*C, *phb*A, *phb*B and *ppn*K genes were calculated as a ratio of 16S rRNA gene expression. The results are reported as the means of data from three experiments

In order to investigate the effect of PHB accumulation on gene transcription, RT-PCR was performed. In this experiment, we selected *argB, argC, argD, argF, argG, argH* and *argJ* gene as the experimental target since they directly involved in the l-arginine production. We found that the transcription levels of four l-arginine operon genes, *argB, argC, argD* and *argJ* in *C. crenatum* P1 increased 1.9–3.0 times compared with the parent strain (Fig. 3). Although the transcription of other genes in *C. crenatum* P1 decreased slightly, this result at least proved that the intracellular PHB accumulation enhanced the transcription levels of several l-arginine key synthesis genes. At the same time, the *phb*CAB overexpression in *C. crenatum* P1 caused a dramatic up-regulation of *ppn*K transcription. In *C. crenatum* P2, a similar data were also obtained for the *phb*CAB and *ppn*K gene which an increase in gene expression corresponded to an increase in enzyme activities. Thus, the introduction of the PHB synthesis pathway and NAD kinase overexpression affected the transcription of key genes of the l-arginine biosynthesis pathway. Obviously, the *phb*C and *ppn*K gene under the control of the promoters P*phbC* and *tac*M caused the increased transcription of the two genes. The two genes *phb*B and *phb*A followed the same trend, but it was much less pronounced. Consequensely, l-arginine yield increasing effect in recombinant *C. crenatum* was explained at the transcriptional level.

### TEM imaging

**Fig. 4:**
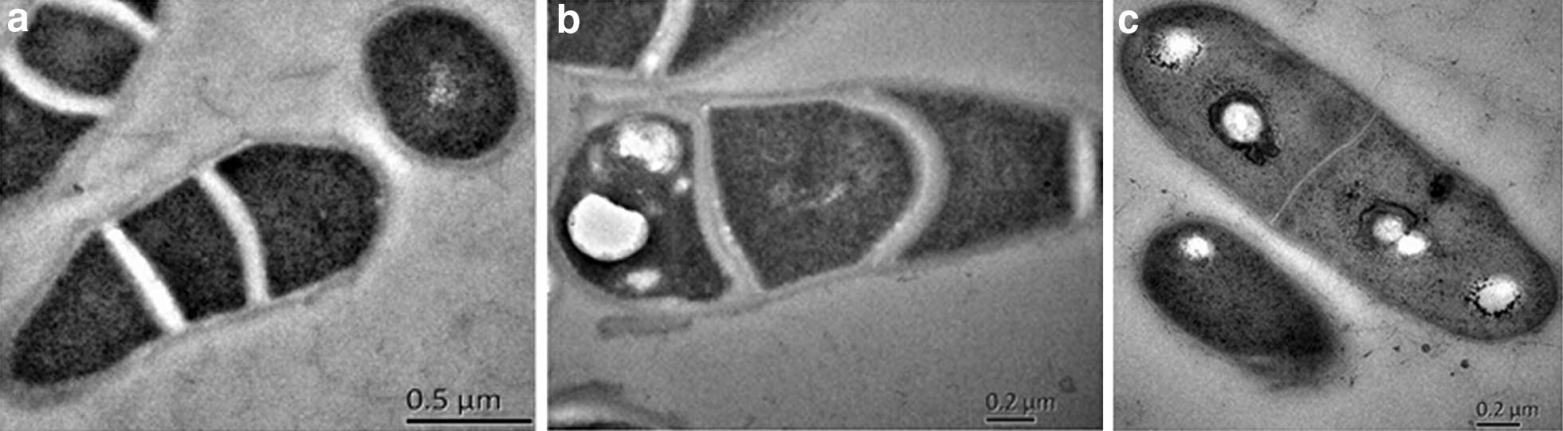
TEM images of *C. crenatum* SYPA 5 and the recombinant *C. crenatum* P1(SYPA 5/*phb*CAB) and P2 (SYPA 5/*phb*CAB-*ppn*K) biomasses. Bacteria appear in *gray* and PHB vesicles in *white* **a** *C. crenatum* SYPA 5; **b** *C. crenatum* P1; **c** *C. crenatum* P2

In order to verify the expression effect of recombinant plasmid pDXW-10-*phbCAB* more directly, *C. crenatum* SYPA 5 and its recombinants were prepared for TEM analysis. The results were exhibited in Fig. 4. From Fig. 4, (a) showed *C. crenatum* SYPA 5 with an extremely small amount of PHB granules; (b) displayed the PHB granules in the recombinant SYPA 5/pDXW-10-*phbCAB*. Due to the PHB gene cluster expressing well in the strain, PHB granules could be seen obviously; thanks to NAD kinase expression in *C. crenatum*, the competition environment of the NADPH was eased in the process of l-arginine and PHB biosynthesis. As it is shown in (c), the PHB existed evidently in the cell, more than that of the strain with PHB synthesis operon only. Thus, we conclude that, the NAD kinase in the recombinant *C. crenatum* was overexpressed.

### Effect of PHB accumulation on l-arginine production by *C. crenatum* P1 in 5–l fermentor

**Fig. 5:**
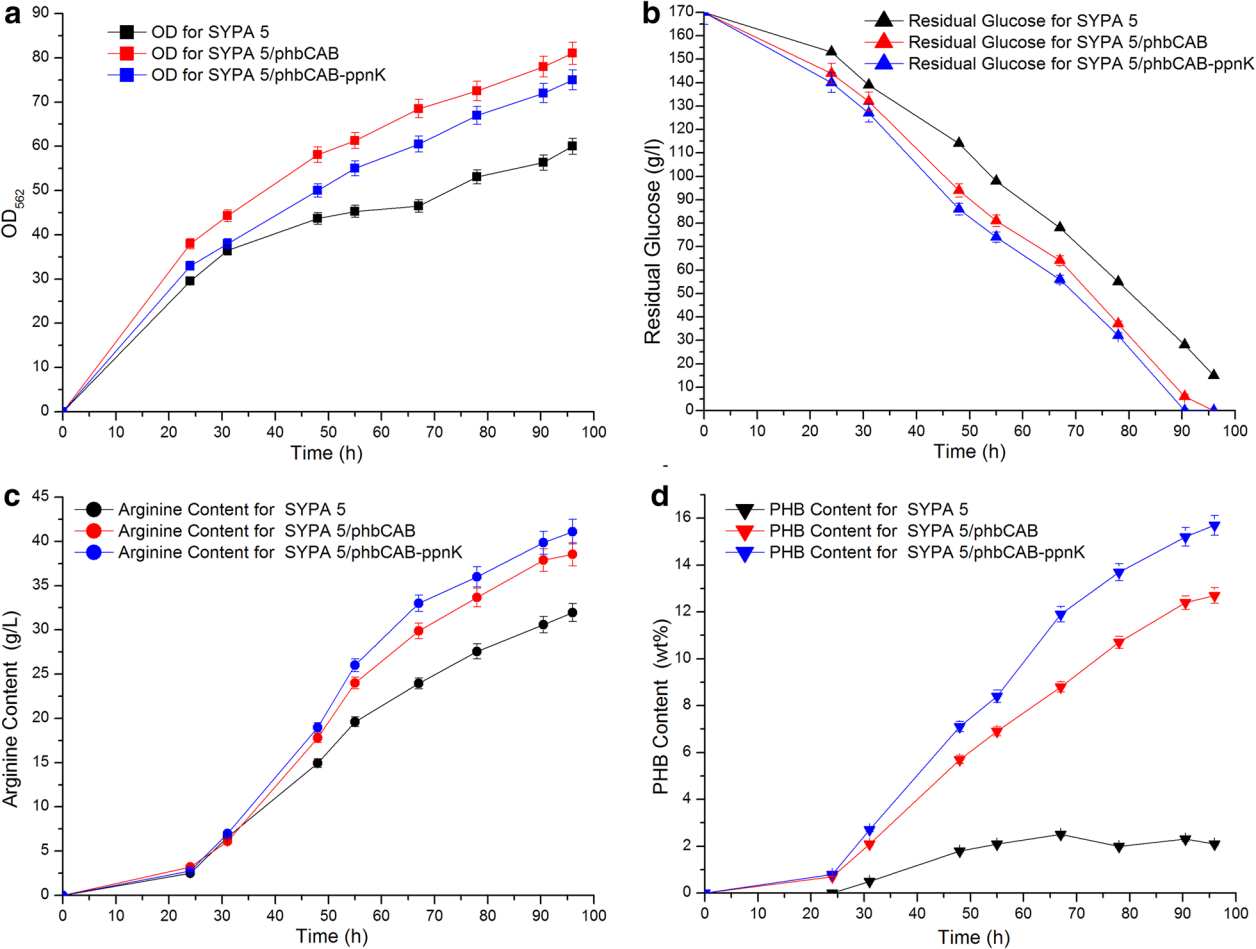
Batch fermentation profile. Comparisons of arginine production between the wild-type *C. crenatum* SYPA 5 and the recombinant *C. crenatum* P1(SYPA 5/*phb*CAB) and P2 (SYPA 5/*phb*CAB-*ppn*K) in fermentation media. **a** OD_562._ (*Black filled square*) *C. crenatum* SYPA 5; (*Red filled square*) *C. crenatum* P1; (*Blue filled square*) *C. crenatum* P2. **b** Residual Glucose. (*Black filled triangle*) *C. crenatum* SYPA 5; (*Red filled triangle*) *C. crenatum* P1; (*Blue filled triangle*) *C. crenatum* P2. **c** l-arginine. (*Black filled circle*) *C. crenatum* SYPA 5; (*Red filled circle*) *C. crenatum* P1; (*Blue filled circle*) *C. crenatum* P2. **d** PHB content. (*Black filled inverted triangle*) *C. crenatum* SYPA 5; (*Red filled inverted triangle*) *C. crenatum* P1; (*Blue filled inverted triangle*) *C. crenatum* P2

To find the effect of PHB accumulation on l-arginine production, the growth of the strains *C. crenatum* SYPA 5 and *C. crenatum* P1 were compared under batch cultivation condition. From Fig. 5, it was easy to find that the PHB content in *C. crenatum* SYPA 5 was low, varied below 3.0 %, while in *C. crenatum* P1, the PHB content increased to 12.7 %, about fourfold, at maximum and more PHB accumulated in the *C. crenatum* P1. The recombinant *C. crenatum* P1 demonstrated a superior ability in growth compared to that of the control *C. crenatum* SYPA 5 with the final OD_562_ reaching 81 at 96 h. However, the glucose consumption of the recombinant strain was more than that of the control strain. Besides these differences, the recombinant strain exhibited similar l-arginine production with the control strain in the early stage of the culture. Notably, the large gap in l-arginine production appeared after 32 h comparing to the control. The final concentration of l-arginine of *C. crenatum* P1 was 38.54 ± 0.81 g/L, increasing by 20.6 % comparing to *C. crenatum* SYPA 5 (31.95 ± 0.68 g/L). Meanwhile, the metabolic intermediate and by-product, α-ketoglutarate, acetate and lactic acid (Table 2) and some other amino acids (Table 3) in batch cultivation were also analyzed to investigate the effect of PHB accumulation on *C. crenatum* metabolism. These data show that, during the fermentation, the formation of some other amino acids except for l-arginine and l-glycine in *C. crenatum* P1 were apparently less than that in the *C. crenatum* SYPA 5. However, the concentration of acetate and lactic acid increased.

**Table 2:** Concentrations of acetate and lactic acid in batch cultivation of *C. crenatum* SYPA 5, P1 (SYPA 5/*phb*CAB) and P2 (SYPA 5/*phb*CAB-*ppn*K)

By-products	Concentration (g/L)				
	24 h	48 h	60 h	84 h	96 h
Acetate					
*C. crenatum* SYPA 5	2.02 ± 0.03	1.74 ± 0.02	1.50 ± 0.01	0.82 ± 0.02	0.74 ± 0.01
*C. crenatum* P1	2.13 ± 0.03	1.81 ± 0.02	1.69 ± 0.02	1.37 ± 0.02	1.18 ± 0.01
*C. crenatum* P2	2.08 ± 0.04	1.88 ± 0.02	1.46 ± 0.02	1.01 ± 0.02	0.94 ± 0.02
Lactic acid					
*C. crenatum* SYPA 5	2.26 ± 0.03	1.80 ± 0.02	1.35 ± 0.02	1.07 ± 0.02	0.86 ± 0.01
*C. crenatum* P1	2.37 ± 0.03	1.97 ± 0.03	1.65 ± 0.02	1.76 ± 0.02	1.95 ± 0.02
*C. crenatum* P2	2.21 ± 0.01	1.78 ± 0.03	1.29 ± 0.02	1.37 ± 0.02	1.50 ± 0.02

Each data represented the average value of three independent measurements

**Table 3:** Production of other related amino acids by *C. crenatum* SYPA 5, P1 (SYPA 5/*phb*CAB) and P2 (SYPA 5/*phb*CAB-*ppn*K)

Amino acids	Concentration (g/L)		
	*C. crenatum* SYPA 5	*C. crenatum* P1	*C. crenatum P2*
Ile	2.62 ± 0.00	2.34 ± 0.00	3.60 ± 0.01
Lys	4.39 ± 0.01	4.09 ± 0.01	5.04 ± 0.02
Glu	1.03 ± 0.02	0.73 ± 0.00	0.51 ± 0.00
Gly	0.30 ± 0.00	0.45 ± 0.00	0.23 ± 0.00
Thr	0.29 ± 0.00	0.19 ± 0.00	0.17 ± 0.00
Val	0.56 ± 0.00	0.42 ± 0.00	0.38 ± 0.00
Orn	0.52 ± 0.00	0.26 ± 0.00	0.26 ± 0.00
Ser	0.21 ± 0.00	0.10 ± 0.00	0.10 ± 0.00

The samples were taken at 96 h of the batch fermentation in 5–l fermentor. Each data represented the average value of three independent measurements

As reported, the expression of PHB synthesis genes, which increased L-glutamate production with 39–68 % in shake flask and 23 % in fermentor, had a positive effect on glutamate production in *C. glutamicum* [18]. PHB also had a global effect on the host in l-tryptophan producing strain and upregulated the transcription of a tryptophan operon, leading to improvement of the l-tryptophan production [17]. Given these results, it was assumed that the introduction of the PHB synthesis pathway into the cells would affect microbial global metabolism, leading to a difference in the formation of some products, such as some kind of amino acids. In this study, due to the exogenous plasmid pDP10 harboring the PHB synthesis genes, *phbCAB*, in *C. crenatum* P1, the recombinant strain grew faster even though the glucose metabolism was enhanced. At the same time, the significant metabolite intermediate for l-arginine production, α-ketoglutarate (from 0.64 ± 0.03 g/L to 0.96 ± 0.02 g/L), had a slight increase flux in the recombinant strain. More bacterial and more metabolic precursor might result in more l-arginine production than that in the parent strain. Moreover, the existence of PHB in the cells might enhance stress resistance of the host and protect the cells, to some degree. Still, more glucose was consumed and conserved in the cell at the cost. It was obvious that the accumulation of PHB affecting the productivity of the long l-arginine fermentation. This might be a major reason why overexpressed *phb*CAB could evolve significant l-arginine yield.

PHB accumulation in the bacteria influenced and regulated the global pathways, including the co-factor level. The NADPH pool in the cells had a slight increase, from 35 ± 2 pmol/OD_562_ to 39 ± 2 pmol/OD_562_, in *C. crenatum* P1 (Table 4), this was likely why more l-arginine was yielded. However, considering that the competitive relationship between PHB and l-arginine production on NADPH, it is interesting to take measures to regulate the cofactor level in the cells in order to see whether any further positive effect on the l-arginine yield exists.

**Table 4:** Concentrations of intracellular NAD^+^, NADH, NADP^+^ and NADPH in *C. crenatum* SYPA 5, P1 (SYPA 5/*phb*CAB) and P2 (SYPA 5/*phb*CAB-*ppn*K)

Strains	Concentration (pmol/OD_562_)				
	NAD^+^	NADH	NADP^+^	NADPH	(NADP^+^ + NADPH)/(NAD^+^ + NADH)
*C. crenatum* SYPA 5	571 ± 42	61 ± 4	161 ± 9	35 ± 2	0.31
*C. crenatum* P1	366 ± 27	70 ± 5	127 ± 8	39 ± 2	0.38
*C. crenatum* P2	163 ± 10	65 ± 4	205 ± 14	71 ± 5	1.21

The samples were taken at 96 h of the batch fermentation in 5–l fermentor. Each data represented the average value of three independent measurements

### Effect of PpnK introduction on l-arginine production

As reported, the impact of *ppnK* overexpression on lysine production was positive in *C. glutamicum* [34], so was isoleucine production in *C. glutamicum* [39] and thymidine production in *E. coli* [33]. In this study, we found that PHB accumulated in recombinant *C. crenatum* P1 and that l-arginine production increased by 20.6 % due to the accumulation of PHB (Fig. 5). Furthermore, NADPH pool played an important role in PHB and l-arginine production. Therefore, it was interesting to see how the extra introduction of NAD kinase into *C. crenatum* P1 affected PHB and l-arginine biosynthesis. In the bacteria, the reducing power [H] was mainly generated by EMP, HMP and TCA cycle while the formation of PHB and l-arginine consumed [H], which keep the oxidation/reduction stateed balanced. The overexpression of PpnK increased the (NADP^+^ + NADPH)/(NAD^+^ + NADH) rate by fourfold in *C. crenatum* P2 comparing to that in *C. crenatum* SYPA 5. As consequence, there was more NADPH supply for PHB synthesis, accounting for 15.7 % of increased PHB at max while 12.7 % in *C. crenatum* P1 (Fig. 5). Excess amount of NAD kinase could be inhibited by the regulation mechanism, like NADP^+^ [43]. In our investigation, two more exogenous genes existed in the cells. Both of these could contribute to the decrease in OD_562_ in *C. crenatum* P2 comparing to that in *C. crenatum* P1. However, the OD_562_ in *C. crenatum* P2 was still above that in *C. crenatum* SYPA 5 and this might be due to the PHB synthesis genes existed (Fig. 5). The concentration of α-ketoglutarate exhibited no much difference between *C.crenatum* SYPA 5 and *C. crenatum* P2 in the early stage but a slight increase in *C. crenatum* P2 after 84 h (data was not shown). Higher growth level and stronger pathway from α-ketoglutarate to glutamate due to more NADPH supply in *C. crenatum* P2 would be why the l-arginine production was higher than that in *C. crenatum* P1, reaching 41.11 ± 2.11 g/L (Fig. 5). In addition, less production of some other amino acids in *C. crenatum* P2 but Ile and Lys, which were quiet NADPH-dependent and increased by 53.8 and 23.2 % comparing to that of *C. crenatum* P1, represently, also contributed to the high production of l-arginine (Table 3). With respect to the concentration of by-products, acetate and lactic acid in *C. crenatum* P2 were lower than those in P1 because of the enhancement of the ratio of (NADP^+^ + NADPH)/(NAD^+^ + NADH) due to the overexpression of *ppn*K (Table 2). All of these could also explain why more l-arginine was produced. The concentration of NADPH was increased by onefold resulted from PpnK overexpression in *C. crenatum* P2 comparing to *C. crenatum* SYPA 5 (Table 4), which enhanced the metabolism, to some degree, especially in those NADPH-dependent pathways. That would be the reason why the glucose consumption rate in *C. crenatum* P2 was faster than both of that in *C.crenatum* SYPA 5 and *C. crenatum* P1 (Fig. 5). Accordingly, increasing the glucose consumption resulted in a higher glucose-to-l-arginine conversion. The *C. crenatum* P2 showed the slight improvement in the glucose-l-arginine conversion with a 14.2 % increase, compared to the parent strain *C. crenatum* SYPA 5(24.1 g Arg/100 g Glc vs. 21.1 g Arg/100 g Glc). Besides improving the conversion of glucose to l-arginine, employing the PHB synthesis pathway led to an increase of glucose to PHB. The intracellular PHB content began to sharply increase to 15.7 % upon the overexpression of *phb*CAB operon in the culture broth of *C. crenatum* P2. Taken together, the more glucose utilization have been achieved by introducing PHB synthesis pathway, together with up-regulation of coenzyme level by overexpressing NAD kinase in *C. crenatum* SYPA 5.

## Conclusions

In conclusion, in *C. crenatum* recombinants harboring the *phb*CAB cluster, more glucose was consumed and conserved in the cell at the cost. The existence of PHB in the cells might enhance stress resistance of the host and protect the cells. Obviously, by employing the PHB synthesis pathway, the l-arginine productivity was increased during the late stage of high-yield l-arginine fermentation. Therefore, it was optimistic that the accumulation of PHB affecting the productivity of the long-time l-arginine fermentation. Meanwhile, the high-yield l-arginine recombinant *C. crenatum* P2 was constructed by overexpressing the NAD kinase encoding gene *ppn*K into *C. crenatum* P1. Overexpressing the NAD kinase also enhanced these effects. Furthermore, batch cultivation of the engineered *C. crenatum* revealed that it could accumulate both extracellular l-arginine and intracellular PHB simultaneously. With those strategies, the recombinant *C. crenatum* with PHB accumulation and NAD kinase overexpression could increase the concentration of NADPH in the coenzyme pool of the cell and serve as high-efficiency cell factories for l-arginine production.

## Methods

### Bacterial strains and plasmids

All the bacterial and plasmids used in the study are listed in Table 5.

**Table 5:** Strains and plasmids used in this study

Strains and plasmids	Relevant characteristics	References
*Corynebacterium crenatum* SYPA 5	l-Arginine production bacterium	[12]
*C. crenatum* P1	Derived from *C. crenatum* SYPA 5, Harboring pDP10	This study
*C. crenatum* P2	Derived from *C. crenatum* SYPA 5, harboring pDPP10	This study
pBHR68	pBluescript SK-derivative, containing the entire *phbCAB* operon of *Ralstonia eutropha* H16	[44]
pDXW-10	*E. coli*–*C. crenatum* shuttle expression vector, Km^r^	[42]
pDP10	Derived from pDXW-10, harboring *phbCAB* operon	This study
pDPP10	Derived from pDP10, harboring *phbCAB* operon and *ppnK* gene	This study
pDP01	Derived from pDXW-10, harboring *ppnK* gene	This study

### Construction of plasmids

The *phbCAB* operon was obtained by double-digesting the plasmid pBHR68 with *Eco*R I and *Bam*H I. It was then ligated into the *E. coli*-*C. crenatum* shuttle expression vector, pDXW-10, to generate the recombinant plasmid pDP10. The native promoter of the *phb*C gene was deleted and the native ribosome binding site (RBS) was changed with a consensus RBS sequence AAAGGAGGGAAATC of highly expressed gene. To construct pDPP10, two steps were done. Firstly, *ppnK* gene from *C. crenatum* SYPA 5 was amplified by primers (5′-ATTTGCGGCCGC AAAGGAGGGAAATC ATGACTGCACCCACGAA-3′) and (5′-CCCAAGCTT TTACCCCGCTGACCTGG-3′) using *C. crenatum* SYPA 5 genome as a temple (the two primers above was designed using the *ppn*K gene of *C. glutamicum* ATCC 13032). Then the PCR product was digested by *Not*I and *Hin*dIII and inserted into *Not* I-*Hin*d III sites of pDXW-10. All of these resulted in the pDP01. Secondly, the pDP01 was used as the temple and the *tac*M-*ppn*K site was amplified by primer 5′-CCCAAGCTTTTACCCCGCTGACCTGG-3′ and 5′- CCCAAGCTTTCGGAAGCTGTGGTATGG-3′. The PCR product was inserted into pDP10 with the *Hin*d III digestion site after dephosphorylation using CIAP. Both of the pDP10 and pDPP10 were transferred into *C. crenatum* SYPA 5, generating *C. crenatum* P1 and *C. crenatum* P2.

### Cultivation medium and conditions

LBG medium (LB with 0.5 % glucose) was used for seed culture preparation supplemented with appropriate antibiotic (kanamycin, 25 mg/L). The fermentation medium contained the following (g/L): glucose 170, (NH_4_)_2_SO_4_ 20, yeast extract 12, MgSO_4_·7H_2_O 0.5, KCl 1, KH_2_PO_4_ 1.5, FeSO_4_·7H_2_O 0.02, MnSO_4_·H_2_O 0.02 (pH 7.0).

The seed cultures were incubated at 30 °C in LBG for 24 h at 160 rpm on a rotary shaker and then 125 ml seed culture was incubated into the fermentation medium (inoculated with 5 % v/v seed culture)for the 5–l fermentor (BIOTECH-5BG, Baoxing Co., China) with a working volume of 2.5–l. The culture condition was set at 30 °C and pH 7.0 under the 600 rpm agitation speed. The pH was controlled automatically by addition of 50 % ammonia water. Temperature was also adjusted automatically by the fermentor.

### RNA preparation and transcriptional analysis

Samples for RNA preparation were cultivated for 24 h in LBG in shake flask at 30 °C. Total cellular RNA was extracted using the RNA simple Total RNA Kit (TIAN GEN, China). Reverse transcription was carried out with the PrimeScript™ RT reagent Kit (TaKaRa, China) according to the instructions of the manufacturer. The mRNA levels were determined by semi-quantitative reverse transcription (q) RT-PCR using SYBR green PCR master mix (ABI 7000; Applied Biosystems, CA). The 16S rRNA gene was used as an endogenous control. For qRT-PCR, 1/20 of each RT-PCR product was used as the template for DNA amplification, using specific primer pairs for each gene. The results of the reactions were processed using specific software (ABI Prism 7000 SDS software). The RT-PCR measurement was repeated three times for each sample. The *argB, argC, argD, argF, argG, argH,argJ, phbC, phbA, phbB* and *ppnK* gene transcript primers were listed in Table 6.

**Table 6:** Primers of RT-PCR used in this study

Primers	Nucleotide sequence (5′–3′)
*argB*	
-F	TCGGTGTTGCTGGAGCTTT
-R	TTCCCCATCCTTGTCGTCTT
*argC*	
-F	AGTCCTTGTTACCTCCGCAATC
-R	CTGCTGCCTCATCAAAACCA
*argD*	
-F	CTTGATGTTGGGCGTGGT
-R	GCGTCTGCGATTTCTTCGT
*argF*	
-F	ACCACACCTTTCGTTCCTTACC
-R	AGGCGGTTTTCTGCTTCATC
*argG*	
-F	TCTCGTGGGCATCAAGTCC
-R	TGACATCTTCCAAAGCCTCGT
*argH*	
-F	AATCATGCCGCAGAAGAAGAA
-R	GTCAAGGTGGAAACCAAACCA
*argJ*	
-F	GTGAAGGTGAGCCGAGAGAAC
-R	ACCATTACACGCATTAGCATTACC
*phbC*	
-F	GCGTTCTACCTGCTCAATG
-R	GATTGGTGGCAAGGAAGTT
*phbA*	
-F	CAAGGAATACGGCATCACA
-R	CGAACTCGTCGGTCTTGAA
*phbB*	
-F	GACGAGATGTTGACGATGC
-R	GACGAGATGTTGACGATGC
*ppnK*	
-F	GTCTGACTCACTTGAAGAGGC
-R	GCAACCAAAGGAAGCAAC

### Sample preparation for TEM imaging

For all different samples for TEM imaging were prepared as reported [45]. Bacteria were fixed with glutaraldehyde 2 % and paraformaldehyde 2 % in a buffer of sodium cacodylate 0.1 mol/L (pH 7.4) and then post-fixed in 1 % buffered osmium tetroxide. The bacteria was then completely dehydrated with ethanol at room temperature and then embedded in epoxy resin (polymerization at 60 °C for 48 h). Ultrathin sections of 50 nm thicknesses were cut with a diamond knife, deposited on copper grids (mesh 200) and then stained with uranyl acetate aqueous solution (20 min) and lead citrate (5 min). Samples were observed at 80 kV with HITACHI H-7650 equipped with a CCD camera.

### Analytical methods

Bacterial growth was monitored by measuring the optical density (OD) at 562 nm using the spectrophotometer, and the dry cell weight (DCW) was determined by a pre-calibrated relationship (1 OD = 0.375 g L^−1^ DCW). Glucose concentration in the media assayed using a glucose analyzer (Biosensor SBA-50, Shandong, China). The enzyme activities of PhbC, PhbA, PhbB and PpnK were detected by the methods reported after the cells were washed twice with 0.1 M Tris–HCl (pH 7.5) and then disrupted by sonication [21, 37]. The concentration of total cellular proteins was determined by the Modified Bradford Protein Assay Kit (Sangon Biotech, Shanghai, China). Bacterial were harvested by centrifugation at 1000 rpm for 10 min, washed twice by distilled water, and then evaporated overnight for the next PHB content detection. PHB content was then tested by gas chromatograph (GC) after methanolysis of lyophilized cells in chloroform [46]. Extracellular l-arginine and other related amino acids were measured by an Agilent 1100 HPLC. The metabolic intermediates or by-products in batch cultivation were analyzed by HPLC (SHIMADZU LC-20A) equipped with an ion exchange column (Aminexs HPX-87H, 7.8 × 300 mm, BioRad). The mobile phase was 5 mM H_2_SO_4_ pumped at a flow rate of 0.5 ml min^−1^. NADPH and NADP^+^ concentration were detected using NADP^+^/NADPH Quantification Colorimetric Kit (BioVision Incorporated, USA). All of the measurements, particularly the most important state variables, were measured in three parallels.

## Additional file

10.1186/s12934-016-0414-x Assay of enzyme activities of crude PpnK. Samples were taken at 24 h of the shake flask using LBG culture. NAD kinase encoding by *ppnK* in *C. crenatum* contained ATP-NAD^+^ kinase, ATP-NADH kinase, PolyP-NAD^+^ kinase and PolyP-NADH kinase. Each data represented the average value of three independent measurements.

## Abbreviations

PHB: Poly-β-hydroxybutyrate
PhbC: PHB synthase
PhbA: β-Ketothiolase
PhbB: NADPH-dependent acetoacetyl-CoA reductase
ArgC: N-acetylglutamate 5-semialdehyde dehydrogenase
PpnK: NAD kinase
LBG: LB with 0.5 % glucose
TEM: Transmission electron microscope
DCW: Dry cell weight
