# Supplementary material for: Effect of Polyhydroxybutyrate (PHB) storage on l-arginine production in recombinant Corynebacterium crenatum using coenzyme regulation
Source: Microb Cell Fact. 2016 Jan 19;15:15. doi: 10.1186/s12934-016-0414-x (PMC4719700; doi:10.1186/s12934-016-0414-x)
Supplement: Supplementary file 1 — 10.1186/s12934-016-0414-x Assay of enzyme activities of crude PpnK. Samples were taken at 24 h of the shake flask using LBG culture. NAD kinase encoding by ppnK in C. crenatum contained ATP-NAD+ kinase, ATP-NADH kinase, PolyP-NAD+ kinase and PolyP-NADH kinase. Each data represented the average value of three independent measurements. [file 12934_2016_414_MOESM1_ESM.doc]

**Table - Assay of enzyme activities of crude PpnK**

| Strains | Specific enzyme activities (U/g) | | | |
| --- | --- | --- | --- | --- |
|  | ATP-NAD^+^ kinase | ATP-NADH kinase | PolyP-NAD^+^ kinase | PolyP-NADH kinase |
| *C. crenatum* SYPA 5 | 0.57±0.03 | 0.06±0.00 | 0.16±0.01 | 0.02±0.00 |
| *C. crenatum* P1 | 0.59±0.04 | 0.06±0.00 | 0.17±0.01 | 0.03±0.00 |
| *C. crenatum* P2 | 82.29±0.38 | 2.06±0.03 | 2.99±0.02 | 0.13±0.00 |

Samples were taken at 24 h of the shake flask using LBG culture. NAD kinase encoding by *ppnK* in *C. crenatum* contained ATP-NAD^+^ kinase, ATP-NADH kinase, PolyP-NAD^+^ kinase and PolyP-NADH kinase. Each data represented the average value of three independent measurements.
